# CD40L and Its Receptors in Atherothrombosis—An Update

**DOI:** 10.3389/fcvm.2017.00040

**Published:** 2017-06-20

**Authors:** Nathaly Anto Michel, Andreas Zirlik, Dennis Wolf

**Affiliations:** ^1^Faculty of Medicine, Department of Cardiology and Angiology I, Heart Center Freiburg, University of Freiburg, Freiburg, Germany

**Keywords:** CD40L, CD40 signaling, Mac-1, cardiovascular diseases, atherosclerosis, thrombosis, inflammation

## Abstract

CD40L (CD154), a member of the tumor necrosis factor superfamily, is a co-stimulatory molecule that was first discovered on activated T cells. Beyond its fundamental role in adaptive immunity—ligation of CD40L to its receptor CD40 is a prerequisite for B cell activation and antibody production—evidence from more than two decades has expanded our understanding of CD40L as a powerful modulator of inflammatory pathways. Although inhibition of CD40L with neutralizing antibodies has induced life-threatening side effects in clinical trials, the discovery of cell-specific effects and novel receptors with distinct functional consequences has opened a new path for therapies that specifically target detrimental properties of CD40L. Here, we carefully evaluate the signaling network of CD40L by gene enrichment analysis and its cell-specific expression, and thoroughly discuss its role in cardiovascular pathologies with a specific emphasis on atherosclerotic and thrombotic disease.

Cardiovascular disease is the major cause of mortality worldwide and is predominantly caused by atherosclerosis, a chronic narrowing of middle sized and large arteries by the buildup of atherosclerotic plaques (1). Subclinical atherosclerosis precedes its potentially life-threatening complications, including acute arterial thrombosis, myocardial infarction, and stroke (2). In the past few decades, it has been well established that inflammatory cues critically fuel the initiation, progression, and complication of atherosclerosis by promoting accumulation of inflammatory leukocytes in the plaque and by driving inflammatory gene expression both systemically and in the atherosclerotic lesion (3). In addition, inflammation exacerbates cardiovascular disease risk factors such as obesity, hypertension, dyslipidemia, and insulin resistance. Therefore, the modulation of inflammatory pathways has been proposed to be a powerful therapeutic strategy against cardiovascular disease (4). Because inflammation is involved in a variety of physiological processes, including host defense, wound healing, hemostasis, and regeneration, the search for pathways and effector molecules that specifically enhance abnormal and dysregulated inflammation has become a major goal. The tumor necrosis factor (TNF) receptor superfamily (TNFRSF) comprises a class of 29 receptors with structural similarities and overlapping functions that can selectively bind one or more of the 19 members of the TNF (ligand) superfamily (TNFSF). These receptor/ligand pairs regulate survival and activation of immune cells and drive the expression of genes that modulate inflammation, immunity, and autoimmunity (5). The ability to modulate inflammation by targeting members of the TNF superfamily is best illustrated by the clinical inhibition of TNF-α, which is now considered to be a hallmark of immunotherapy and anti-inflammatory therapy in a variety of chronic inflammatory pathologies (6).

## CD40L—More than a Co-Stimulator of B Cells

**Figure 1:**
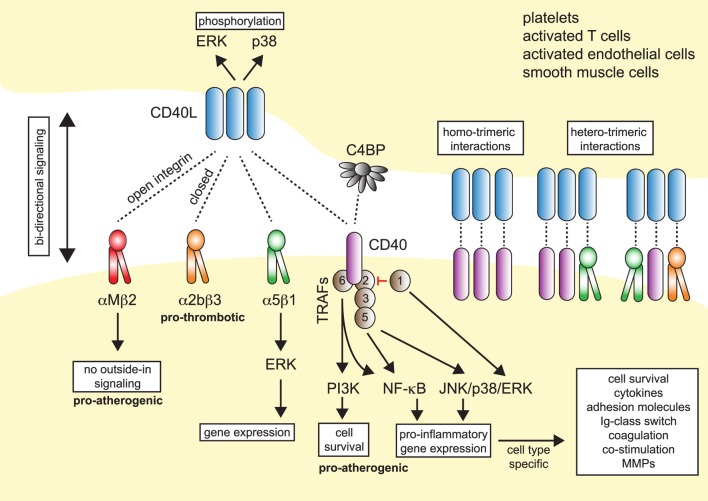
Model of CD40L–receptor interactions. Membrane-bound CD40L forms biologically active trimers that can interact with one of its known receptors, the integrins αIIbβ3, α5β1, αMβ2 (Mac-1), or its “classical” receptor CD40. While binding to CD40 occurs statically, binding to integrins can be enhanced by activation-induced conformational changes of both subunits that expose the ligand-binding site (inside-out signaling). Binding to Mac-1 occurs predominantly through the activated integrin, binding to α5β1 requires no previous cell activation and switching into the open, high-affinity conformation. Binding to αIIbβ3 usually occurs through the open conformation, but exact binding properties to CD40L have not been investigated in detail. Besides CD40L, CD40 can also interact with the complement-factor C4b-binding protein (C4BP). Binding of CD40L to one of its receptors induces downstream signaling events, except for Mac-1, for which CD40L serves as biased agonist without induction of outside-in signaling. Binding to α5β1 causes an activation of MAP-kinase signaling pathways, ligation to CD40 causes activation of mitogen-activated protein kinase (MAPK)-, phosphoinositide 3-kinase (PI3K)-, and nuclear factor-κB (NF-κB)-signaling events and subsequent pro-inflammatory gene expression. The consequences of CD40 signaling depend on the target cell type. On the contrary, ligation of CD40L to a receptor induces bidirectional signaling events in CD40L-bearing cells, e.g., T cells, B cells, or platelets, possibly by induction of MAPK signaling cascades. Binding of CD40L to its ligands can occur in a homotrimeric fashion, where a trimer of CD40L binds three monomers of CD40, or in a heterotrimeric fashion, where each monomer (of a trimer of CD40L) can bind to different receptors, which was demonstrated for CD40, αIIbβ3, and α5β1.

CD40L (also known as CD154, gp39, TRAP, TBAM) is a member of the TNF superfamily that was first identified on activated T cells, where it interacts with CD40 receptor on B cells to induce B cell activation, proliferation, and IgG-class switching during co-stimulation (7–10). Mutations of the CD40L gene (*Cd40lg*) were identified as the cause of the human X-linked immunodeficiency hyper IgM-syndrome (XHIM), a condition characterized by a loss of T cell-dependent humoral immunity and specific IgG antibodies (11). Apart from the 33 kDa full-length version of CD40L that forms trimeric complexes on the cell surface, a truncated 18-kDa version that lacks the cytoplasmic tail, the transmembrane domain, and parts of the extracellular domain is generated by shedding membrane-anchored CD40L by matrix metalloproteinases (MMPs) and certain disintegrin metalloproteinases (ADAMs) (12–14) (Figure 1). This soluble fraction of CD40L (sCD40L) can be detected in the blood circulation, but it also forms multimeric complexes with the membrane-anchored full-length version of CD40L on the cell surface (15). The biological activity of CD40L increases with a higher multimeric organization (16, 17), and it has previously been described that sCD40L is biologically less active than membrane-bound CD40L (16–21). This may in part be caused by an incomplete formation of sCD40L trimers, as sCD40L lacks the transmembrane domain and parts of the extracellular domain that can support trimerization (16, 17). However, the definitive structural organization of sCD40L is still under debate, as trimers of CD40L have been observed to form through interactions independent of the trimerization domain. Accordingly, the spontaneous formation of monomers, dimers, and trimers of sCD40L are detected in the blood circulation (18, 22, 23).

**Figure 2:**
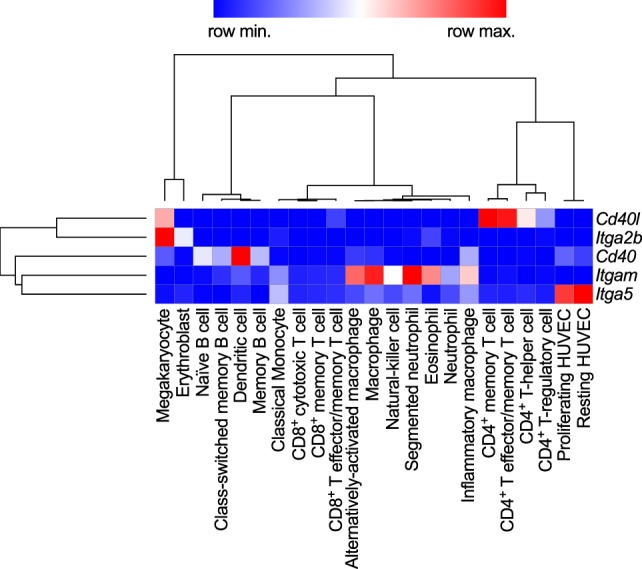
Gene expression patterns of CD40L and its receptors in human immune cell types. Baseline gene expression of different human immune cell types quantified by RNAseq was extracted from the Protein Expression Atlas of the European Bioinformatics Institute (EMBL-EBI) (33). Expression values were retrieved as FPKMs, underwent hierarchical clustering and normalization as row scores by Morpheus (Broad Institute). Gene names are encoding for the proteins as follows: *Cd40lg*: CD40L; *Itgb2*: integrin subunit α2b (CD41); *Cd40*: CD40 receptor; *Itgam*: integrin subunit αM (CD11b); *Itga5*: integrin subunit α5 (CD49e).

Levels of sCD40L have been proposed as biomarkers of atherothrombosis (24, 25). While it was originally perceived that the expression of CD40L is restricted to T cells, numerous studies have shown that a broad range of hematopoietic cells and vascular/stromal cells can express CD40L in an inducible fashion. Cell types expressing CD40L include T cells, B cells, basophils, eosinophils, monocytes, macrophages, Kupffer cells, natural killer (NK) cells, platelets, mast cells, and dendritic cells (DCs), as well as endothelial cells (ECs), smooth muscle cells (SMCs), and epithelial cells [reviewed in Ref. (7)]. Among hematopoietic cells, gene expression of CD40L is highest in T cells and megakaryocytes, the progenitors of platelets in the bone marrow (Figure 2). The detection of CD40L on cells residing in the atherosclerotic plaque—including ECs, macrophages, foam cells, and SMCs—proposed that CD40L contributes to lesion development and inflammation in atherosclerosis (26, 27). Indeed, work for over two decades has identified CD40L as both a pro-inflammatory surface molecule and soluble cytokine (sCD40L), driving expression of other cytokines, chemokines, adhesion molecules, extracellular matrix-degrading enzymes, and mediators of cell survival. Furthermore, CD40L functions as an adhesion receptor that promotes cell recruitment as well as physical interactions between platelets. A functional involvement of CD40L has been shown in several inflammatory and autoimmune pathologies, including arthritis, nephritis, organ rejection, autoimmune diabetes, inflammatory bowel disease, and systemic lupus erythematosus (SLE) (28–32). Traditionally, CD40L was believed to interact solely with CD40. Recent evidence, however, demonstrates the existence and functional participation of alternative receptors. Here, we will review the functional role of CD40L and its receptors in cardiovascular pathologies.

## Receptors for CD40L

Up until now, four different receptors have been identified for CD40L: the three integrins αMβ2 (Mac-1), αIIbβ3, and α5β1, and the “classical” receptor CD40. These are distinguishable by their cellular expression patterns, signaling events, and functional consequences (summarized in Figure 1).

### CD40

CD40, member of the TNF receptor superfamily, is a 48-kDa type I transmembrane protein. In addition to B cells, CD40 is expressed constitutively and in an inducible fashion by T cells, DCs, monocytes, platelets, macrophages, SMCs, ECs, and fibroblasts (34–36). Human CD40 gene expression is highest in DCs and intermediate in B cells and megakaryocytes, i.e., platelets (Figure 2). Its expression is regulated by inflammatory cues, including TNF-α, IL-1, IFN-γ, CD40L, and others (7, 21). Of all receptors that ligate CD40L, CD40 has been reported to exhibit the highest affinity for monomeric CD40L, around ~0.5–7nM (37, 38), but higher affinities are suspected for the interaction with trimeric CD40L (39) (Table 1). Whether monomeric sCD40L can act as a competitive antagonist for membrane-bound CD40L is not known, but the biological activity for multimeric, membrane-bound CD40L is higher than that of monomeric CD40L (16–21, 40). Upon binding by CD40L, CD40 monomers on the cell surface are clustered in trimers, a process that is thought to occur more frequently in lipid rafts and by ligation with CD40L itself (41, 42). Consequently, tumor necrosis factor receptor-associated factors (TRAFs), a group of intracellular adapter proteins that also associate with TNF receptors, toll-like receptors (TLRs), and IL-1 receptors, are recruited to the cytoplasmic domain of CD40 (43), where they activate canonical and non-canonical pathways that can result in an engagement of nuclear factor-κB (NF-κB), MAPKs, and phosphoinositide 3-kinase (PI3K), as well as phospholipase Cγ (44). While TRAF-2/3/5/6 activate downstream signaling, TRAF-1 inactivates TRAF-2 by direct binding and is considered an inhibitory TRAF (45). Biological effects of CD40 ligation include cell survival, proliferation, and inflammatory cytokine and chemokine expression (29, 46, 47). CD40 can have distinct effects on different cell types: ligation of CD40 on B cells induces proliferation, activation, and an IgG-class switch (10), while ECs and fibroblasts increase expression of the adhesion factors VCAM-1, ICAM-1, and E-selectin (7). SMCs and ECs release MPP-1, -2, -3, and -9, which are molecules involved in the destabilization of atherosclerotic plaques (7, 48). In contrast, CD40 seems to have an inhibitory role on T cells, as it prevents cytokine release and T cell activation (49).

**Table 1:** Binding properties of CD40L’s receptors.

Receptor	Binding residues (CD40L)	Binding residues (receptor)	Affinity (nM)
CD40	Y^145^, R^203^ (50, 51)	Y^82^, D^84^, N^86^ (51)	~0.5–7 (37, 38)
	K^143^ (51)	E^74^, E^117^ (52)	
	Y^146^, Q^220^ (52)		
	E^129^, S^132^, T^134^, E^142^ (53)		
αIIβ3	D^117^ (54)	unknown	~30 (54, 55)
Mac-1	Y^145^, R^203^ (50)	E^162^-L^170^ (56)	~200 (56)
α5β1	N^151^, Q^166^ (50)	unknown	~120 (55)

Apart from CD40L, CD40 has also been reported to bind the complement-associated C4b-binding protein (C4BP), an interaction that occurs on a separate site from the CD40L binding epitope (57), although C4BP binding may partially be mediated by cross-binding to CD40L (58). B cells stimulated with C4BP show enhanced proliferation, adhesion receptor expression, and an IL-4-dependent IgE-class switch.

### αMβ2 (Mac-1)

Mac-1 (CD11b/CD18) is a member of the β2-integrin family and a heterodimer of the αM (CD11b) and β2 (CD18) integrin subunit. Mac-1 is predominantly expressed on myeloid cells, such as monocytes, macrophages, and neutrophils, but is also present on NK cells, and, to a smaller extent, on some B cell subsets (B1); however, the highest mRNA abundance of the CD11b αM subunit (*Itgam*) is confined to neutrophils and macrophages (Figure 2). Mac-1 is required for the firm adhesion and slow rolling of leukocytes on ECs (59, 60). Mac-1 interacts with a broad repertoire of different ligands, including C3bi (61), ICAM-1 (59), fibrinogen (62), fibronectin (63), vitronectin (63), heparin (64), GPIbα (65), RAGE (66), endothelial protein C receptor (EPCR) (67), and others (68). We have recently shown that Mac-1 interacts with CD40L by utilizing a binding site on the major ligand-binding I-domain within the αM subunit (E^162^-L^170^), which is distinct from other ligand-binding sites and, thus, is highly selective for CD40L (56, 69). Binding of CD40L by Mac-1 is enhanced by the open, high-affinity conformation of the integrin (50), suggesting that Mac-1/CD40L binding can be regulated by integrin inside-out signaling (70). Peptide inhibition studies have revealed that the CD40L/Mac-1 interaction primarily serves as an adhesive receptor–ligand pair: CD40L expressed on activated ECs binds to Mac-1 on rolling leukocytes to allow their firm adhesion (26, 56, 71). Whether Mac-1 binds to a specific binding site on CD40L is controversial; while Mac-1 binding does not compete with binding of CD40L to CD40 in competitive binding assays (56), a mutant version of CD40L, which lacked the binding site for CD40, also abolished Mac-1 binding (50). These findings suggest that common residues in CD40L involved in the binding of Mac-1 and CD40 exist (Table 1). The main endothelial receptors for Mac-1 include ICAM-1, CD40L, RAGE, and EPCR. In tissue-resident macrophages, where Mac-1 is highly expressed (71, 72), ligation of the integrin promotes cell activation, inflammatory gene expression, and participates in phagocytosis (71–73). While most Mac-1 ligands induce cell activation upon binding—a process referred to as outside-in signaling—CD40L serves as biased agonist that can bind to the integrin without inducing cellular activation (68). Mac-1 is required for many physiological and pathogenic processes involved in inflammation, host defense, and wound healing (72). Inhibition of Mac-1 reduces atherosclerosis (69), neointima formation (74, 75), and thrombotic glomerulonephritis (76) in mice. In humans, a mutation of the β2-subunit, which affects the functionality of Mac-1, LFA-1, and CD11c, is responsible for an immune deficiency known as leukocyte adhesion deficiency (77).

### α5β1

The integrin α5β1 (VLA-5) is a heterodimer of the integrin subunits α5 (CD49e) and β1 (CD29). It serves as a primary receptor for fibronectin and vitronectin through an RGD sequence in both molecules (78, 79). It is expressed in monocytes, macrophages, and ECs. The gene transcript of the α5 subunit (*Itga5*) is most abundant in ECs (Figure 2). In addition, stromal cells such as fibroblasts, chondrocytes, and synovial cells (80) have been reported to express VLA-5. Ligation of the integrin induces MAPK signaling *via* ERK and stimulates cell survival, proliferation, and inflammatory gene expression (81). ECs require functional VLA-5 to attach, spread, and proliferate within the extracellular matrix (82). VLA-5 also interacts with VEGFR-1 (82), angiopoietin-2 (83), and endostatin (84), which may explain its requirement for angiogenesis (85). Leveille et al. identified CD40L as a ligand for α5β1 that unexpectedly—and in contrast to most integrin ligands—binds to the inactivated conformation of the integrin. Binding of CD40L to VLA-5 induced ERK-signaling pathways and IL-8 expression in a human monocytic cell line (80), similarly to the response caused by fibronectin binding to the integrin. Besides a cross talk of cells with the extracellular matrix, the CD40L/VLA-5 interaction was also recently shown to mediate cytokine production and the adhesion of CD40L^+^ T cells with fibroblasts (86) and to inhibit apoptosis in T cells (87). Binding sites of CD40 and α5β1 were mapped to different regions within CD40L, effectively allowing both receptors to simultaneously bind to CD40L trimers (55, 80). Specific inhibitors of the CD40L/VLA-5 interaction and their specific impact on cardiovascular pathologies have not been reported yet.

### αIIbβ3

Expression of the integrin αIIbβ3 (CD49b/CD61, GPIIb3a) and the transcript *Itga2b*, which encodes for the αIIb (CD61) subunit of the integrin, are restricted to the megakaryocyte lineage (Figure 2). In addition, the β3 subunit (*Itgab3*) can form the integrin αVβ3 on platelets and myeloid cells. Platelets express αIIbβ3 on the cell surface, but additional integrin heterodimers are stored in platelet α- and dense granules. These are translocated to the cell surface during platelet activation and degranulation (88). Binding of soluble fibrinogen and von Willebrand factor by αIIbβ3 supports platelet adhesion and aggregation (89). Ligand binding to αIIbβ3 occurs through a site within the α- and β-subunit (90). Clinically, αIIbβ3 is targeted by several platelet aggregation and thrombus blocking drugs, including abciximab, tirofiban, and eptifibatide during cardiac catheterization (89). The main binding site within fibrinogen comprises an Arg–Gly–Asp (RGD) motif that is mimicked by tirofiban and eptifibatide and thus occupies the binding site on αIIbβ3 (91). Ligand binding is regulated by activation-induced conformational changes between the α- and β-subunit and is facilitated by an open, extended confirmation of the integrin, which allows for high-affinity binding (92, 93). Andre et al. established CD40L as a ligand for αIIbβ3 through a KGD-binding motif in CD40L (54). Binding affinity ranges between that of CD40 and Mac-1, VLA-5 (~30nM) (54, 55). sCD40L binding to αIIbβ3 induces signaling events in platelets through p38 and ERK1/2 (50, 54, 94).

### Integrated Model of CD40L–Receptor Interactions

Traditionally, CD40 was believed to be the only receptor for CD40L. Newer evidence, however, has identified at least three alternative receptors of the integrin family: Mac-1, αIIbβ3, and α5β1 (Figure 1). These observations, along with the predominantly cell-specific expression of each of these receptors—αIIbβ3 on platelets, α5β1 on ECs, and Mac-1 on the myeloid cell lineage (Figure 1)—have sparked the notion that CD40L has distinct effects on different cellular subsets: mediating thrombotic and hemostatic properties through its interaction with αIIbβ3, cellular adhesion through Mac-1, immune functions (including B cell activation) through CD40, and activation of ECs through interaction with α5β1. Blocking specific binding sites on one of the participating molecules may enable to therapeutically abrogate distinct effector functions of CD40L. This concept is best illustrated by the M7 peptide that specifically blocks the interaction site for Mac-1 on CD40L without interfering with the CD40L–CD40 interaction (56). CD40L/Mac-1 primarily functions in leukocyte adhesion to the endothelium, but its inhibition by M7 does not affect CD40L’s effect on immunity or thrombosis. Such a concept may also enable inflammation-specific therapy, because (1) CD40L protein levels are upregulated rapidly, either transcriptionally or by mobilization of granule stores, mostly in inflammatory microenvironments and (2) integrins, such as Mac-1 and αIIbβ3, can exist in several conformations (95, 96): Mac-1 and αIIbβ3 primarily bind ligands in the activated conformation, which could be targeted by activation-specific blocking strategies (50, 97). This model is further complicated because CD40L binding to a receptor is not exclusive but instead occurs in a heteromeric manner, where a trimer of membrane-bound CD40L binds to two or more of its ligands simultaneously (Figure 1). For instance, platelets express CD40, αIIbβ3, and α5β1. Recent observations indicate that binding sites on CD40L for at least some of its receptors are distinct and suggest that each monomer (of a trimer) may bind to one of its receptors (21, 50, 55). However, the effect of different affinities between CD40L and its receptors—with the highest affinity recorded for CD40 and the lowest for Mac-1 (Table 1)—on heteromeric binding patterns and subsequent signaling is yet to be determined.

## CD40L in Atherosclerosis

### Clinical Association between Atherosclerosis and CD40L/CD40 Signaling

**Figure 3:**
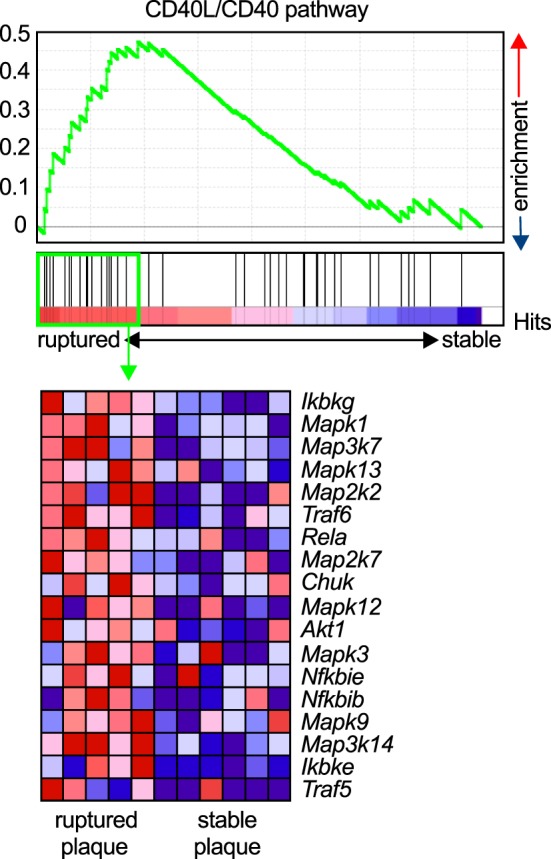
Enrichment of the CD40L/CD40 pathway in unstable human plaques. Gene set enrichment of the core CD40-signaling gene signature (Table 3) was tested on published transcriptomes of human stable and ruptured atherosclerotic plaques from laser-microdissected macrophage-rich regions of carotid endarterectomy specimen. Enrichment was tested between ruptured and stable plaques. The expression of the enrichment-driving genes (lower panel) was blotted on a heatmap and colored by a row-score (blue = lowest, red = highest gene expression per row).

Atherosclerosis is a chronic inflammatory disease of medium to large arteries that is characterized by the buildup of fibro-fatty plaques in the intimal layer of the vessel, which limits blood flow into the distant vascular bed or results in total thrombotic occlusion during myocardial infarction or stroke (1). Atherosclerotic plaque development is critically driven by the accumulation of lipids, mostly low-density lipoprotein, and the subsequent accumulation of immune and inflammatory cells, such as lymphocytes and cells of the myeloid lineage (98). Activation of infiltrating leukocytes by tissue-resident cells or other leukocytes, secretion of pro-inflammatory mediators such as cytokines, chemokines, or matrix-degrading enzymes, and the recruitment of new blood cells through cell adhesion cascades are the functional hallmarks of atherogenesis (3). The expression of CD40L and its classical ligand CD40 on many of the participating cells, including various leukocyte subpopulations, ECs, and SMCs (26), has led to the hypothesis that the CD40L/CD40 dyad may contribute to atherosclerosis. CD40L and CD40 were identified in human atherosclerotic lesions at every developmental stage (27, 34), and CD40 expression in lesional macrophages and SMCs correlates with the stage of atherosclerosis (34). Relatively, CD40L expression is highest in ruptured atherosclerotic lesions (99), likely due to the deposition of CD40L-expressing platelets at the site of the rupture, or enhanced CD40L gene expression in rupture-prone plaques. The latter is supported by gene set enrichment analysis (GSEA) of the core CD40-signaling gene signature found in transcriptomes of both stable and ruptured atherosclerotic plaques from laser-microdissected macrophage-rich regions of human carotid endarterectomy specimens (100). In ruptured plaques, the expression of signaling molecules downstream of CD40, including NF-κB, TRAFs, and MAP kinases, significantly increased compared to stable human plaques (Figure 3), suggesting enhanced CD40 signaling in unstable plaques.

Moreover, plasma levels of soluble CD40L have been proposed as a biomarker for cardiovascular risk; in particular, several studies have established a positive correlation of sCD40L with cardio-metabolic risk factors, such as dyslipidemia, diabetes, and obesity (112–114). The association with cardiovascular disease and outcome, however, is less clear: Lemos et al. found only a minimal correlation of sCD40L plasma levels with hyperlipidemia and no correlation with subclinical atherosclerosis as assessed by coronary calcification and aortic plaque size (114); however, sCD40L levels predicted future cardiovascular events in a case control study in women (115). Other nested case–control studies found a positive correlation with a worsened outcome after myocardial infarction (116), but these findings could not be confirmed in larger cohorts from the same clinical study population (117). It was speculated that the observed clinical associations are caused not by atherosclerosis itself but instead by enhanced platelet release of sCD40L in the setting of pro-thrombotic disease (114). These observations could render sCD40L as a biomarker of thrombotic events and risk, particularly in the setting of myocardial infarction (118–120) and hypercholesterolemia (24).

Data from genome-wide association studies have suggested that certain polymorphisms in the CD40 gene (rs1535045, rs3765459, rs4810485, rs1883832) may be associated with traits of cardiovascular disease, although studies have yielded inconsistent results. rs1535045 and rs3765459 correlated with the calcification score of coronary arteries from diabetic (121) and CVD patients (122). rs4810485, but not rs1535045, correlated with the number of coronary lesions in patients with Kawasaki syndrome (123). The SNP rs1535045 correlates with carotid intima-media thickness, a surrogate marker for atherosclerosis, in patients with rheumatoid arthritis (124). Other studies have also identified a correlation between CVD and rs1883832 (125) and rs4239702 (122). All together, these clinical findings have established a strong association between CD40L/CD40 signaling and cardiovascular disease, particularly in the setting of acute cardiovascular events and unstable atherosclerotic plaques.

### CD40L in Experimental Atherosclerosis: A Controversy

Several studies have demonstrated a functional relevance of CD40L, its receptors, and signaling intermediates in experimental atherosclerosis (overview in Table 2). In 1998, Mach et al. first showed that inhibition of CD40L with a neutralizing anti-CD40L antibody reduced the size of atherosclerotic plaques in *Lldr^−/−^* mice (101). Atherosclerotic plaques of anti-CD40L-treated mice showed a marked reduction of lipid-positive areas, as well as a reduction of macrophage and T cell markers in immunohistochemistry—features that are linked to a stable, and therefore less likely to rupture, atherosclerotic plaque in humans. The observation that leukocyte recruitment was dampened after CD40L blockade was explained by the authors with a decreased expression of the adhesion molecule VCAM-1, which could hinder the recruitment of these cells to the atherosclerotic lesions. Lutgens et al. later confirmed that *Apoe^−/−^* mice treated with an anti-CD40L antibody possessed a stable plaque phenotype—rich in collagen and less populated by macrophages and T-cells. Mechanistically, these effects were explained by enhanced TGF-β signaling (103); however, lesion size was not changed by the anti-CD40L treatment in this study. In another study, CD40L-knockout mice on an *Apoe^−/−^* background were not protected from *de novo* lesion formation but showed a reduction of established atherosclerotic lesions and features of plaque stability (102). Another study in *Ldlr^−/−^* mice with established atherosclerotic lesions showed that the treatment with a blocking anti-CD40L antibody protected from further disease progression, although it did not induce plaque regression (104). While these studies have uniformly established that CD40L affects the cellular and extracellular composition of the atherosclerotic plaque, the impact of CD40L on the size of atherosclerotic lesions remains controversial; some studies show that blocking CD40L (by genetic knockouts or antibodies) decreases lesion size (101, 104, 105), while others show that lesion size remains unaffected (102, 103). This disparity in findings could likely be attributed to the different knockouts, genetic backgrounds (*Apoe^−/−^* vs. *Ldlr^−/−^*), and diet regimens (high-cholesterol diet vs. standard chow diet) tested. In addition, it remains unclear as to whether the blocking antibodies tested would be able to equally target all cell types, as cells within atherosclerotic plaques are less likely to be exposed to the same concentration of antibodies as circulating cells are. However, the common finding that CD40L blockade attenuates lesional accumulation of leukocytes, along with the predominant expression of CD40L on cells of the hematopoietic lineage, suggests that CD40L therapies would block leukocyte migration into the plaque. Yet, in bone marrow transplantation studies where CD40L-deficient bone marrow was transplanted into *Ldlr^−/−^* mice, neither lesion size nor the cellular composition in the plaque were changed (105), indicating that stromal cells are more likely to be the cellular source of bioactive CD40L.

**Table 2:** CD40L-associated molecules in experimental atherosclerosis.

Reference	Target molecule		Model	Diet (weeks)	Lesion size	Plaque stability
Mach et al. (101)	CD40L	Anti-CD40L	*Ldlr^−/−^*	12, HCD	Decreased	More stable
Lutgens et al. (102)	CD40L	Global KO	*Apoe^−/−^*	23, chow diet	Decreased est., not early	More stable established lesions
Lutgens et al. (103)	CD40L	Anti-CD40L	*Apoe^−/−^*	12, chow diet (starting at 5 weeks)	No effect	More stable
Lutgens et al. (103)	CD40L	Anti-CD40L	*Apoe^−/−^*	12, chow diet (starting at 17 weeks)	No effect	More stable
Schönbeck et al. (104)	CD40L	Anti-CD40L	*Ldlr^−/−^*	13, HCD (after 13 weeks HCD alone)	Decreased	More stable
Bavendiek et al. (105)	CD40L	Global KO	*Ldlr^−/−^*	16, HCD	Decreased	More stable
Bavendiek et al. (105)	CD40L	T_x_ of *Cd40l^−/−^* BM	*Ldlr^−/−^*	16, HCD	No effect	No effect
Zirlik et al. (69)	CD40	Global KO	*Ldlr^−/−^*	8 and 16, HCD	No effect	n/a
Lutgens et al. (106)	CD40	Global KO	*Apoe^−/−^*	23, chow diet	Decreased	More stable
Lutgens et al. (106)	CD40	T_x_ of *Cd40^−/−^* BM	*Ldlr^−/−^*	26, chow diet	Decreased	More stable
Lutgens et al. (106)	CD40-T6	KI in MHC-II^+^	*Apoe^−/−^*	26, chow diet	Decreased	More stable
Lutgens et al. (106)	CD40-T2/3/5	KI in MHC-II^+^	*Apoe^−/−^*	26, chow diet	No effect	No effect
Missiou et al. (107)	TRAF-1	Global kO	*Ldlr^−/−^*	8 and 18, HCD	Decreased	More stable
Stachon et al. (108)	TRAF-6	T_x_ of *Traf6^−/−^* BM	*Ldlr^−/−^*	18, HCD	No effect	No effect
Polykratis et al. (109)	TRAF-6^fl^	Tie2CreER	*Apoe^−/−^*	10, HCD	Decreased in females	More stable
Polykratis et al. (109)	TRAF-6^fl^	LysMCre	*Apoe^−/−^*	10, HCD	Increased	No effect
Missiou et al. (110)	TRAF-2	T_x_ of *Traf2^−/−^* BM	*Ldlr^−/−^*	18, HCD	No effect	No effect
Missiou et al. (110)	TRAF-5	Global knockout	*Ldlr^−/−^*	18, HCD	Increased	Unstable
Zirlik et al. (69)	Mac-1	Global knockout	*Ldlr^−/−^*	10, HCD	Decreased	More stable
Wolf et al. (56)	CD40L/Mac-1 binding	Peptide	*Ldlr^−/−^*	20, HCD	Decreased	More stable
Yurdagul et al. (111)	α5β1	Inhibitor of fibronectin	*Apoe^−/−^*	8, HCD	Decreased	More stable

### Is CD40 the Atherogenic Counter Receptor for CD40L?

The observation of enhanced thromboembolic complications after CD40L blockade in human lupus-associated glomerulonephritis (126, 127) has fueled the search for alternative strategies to neutralize CD40L(-signaling). Thus, CD40 receptor was proposed as a potential target. We have recently shown that CD40-knockout mice on an *Ldlr^−/−^* background fed with a high fat diet were not protected from *de novo* atherosclerosis (69); however, another study reported that CD40-deficient *Apoe^−/−^* mice on a standard chow diet for 26 weeks developed reduced atherosclerotic lesions with stable features and lowered leukocyte infiltration. In addition, a transplantation of CD40-deficient bone marrow into *Ldlr^−/−^* mice in the same study was atheroprotective (106). The finding that CD40 deficiency did not protect from atherosclerosis in at least one of these two studies has raised the possibility that CD40 may not be an exclusive receptor for CD40L. In line with this speculation, we recently demonstrated that CD40L interacts with the leukocyte integrin Mac-1 (69). Treatment with blocking anti-Mac-1 antibodies (69), depletion of Mac-1 expressing cells (128), and treatment with a specific inhibitor of this interaction—the peptide M7 (56)—protected from *de novo* atherosclerosis in *Ldlr^−/−^* mice on a high fat diet and reduced the number of lesional leukocytes, suggesting that this interaction may be a driver of leukocyte mobilization into the plaque. In the same study, we found that leukocytes were incapable of adhering to CD40L-deficient ECs (56). These results suggest that CD40L is a relevant adhesion factor on ECs. These findings confirm previous observations that CD40L is required for leukocyte accumulation in the plaque (101–105). Whether the CD40L–α5β1 interaction modulates atherosclerosis is currently unknown. Recent evidence has suggested that blocking α5β1 by a peptide mimetic protects from atherosclerotic lesion development, although the compound used was specific for the fibronectin binding site on α5β1, and not for the interaction to CD40L (111).

### CD40-Dependent Signaling Events in Atherosclerosis

CD40 downstream signaling events are carried out through a group of intracellular signaling molecules known as TRAFs, which activate MAPKs and NF-κB. Signaling through the seven known TRAFs is not exclusive for CD40, because TRAFS are also engaged by TNF receptors, IL-1 receptor, or TLRs. It has been shown that ECs engage TRAF-1, -2, -3, -5, and -6 after stimulation with CD40L, which results in increased expression of inflammatory cytokines (129). In atherosclerotic plaques from *Ldlr*-deficient mice, TRAF-1/2/3/5/6 are overexpressed (129). Different TRAFs activate distinct downstream signaling events: for instance, CD40 signaling induces PIK3-dependent signaling events through TRAF-6, while TRAF-2/3/5 activate JNK, p38, and ERK signaling. In an elegant study by Lutgens et al., the specific binding site for TRAF-6 or TRAF-2/3/5 within CD40 was deleted in MHCII-expressing cells in knockin mice. Surprisingly, a deletion for the CD40-TRAF-2/3/5 interaction had no effect on atherosclerosis, while a deletion for CD40-TRAF-6 inhibited atherosclerotic plaque development and lowered the numbers of macrophages and T cells in the plaque (106). The role of TRAF-6, however, needs to be carefully evaluated for cell-type specific effects: Polykratis et al. found that a conditional knockout of TRAF-6 in ECs was atheroprotective by inhibiting NF-κB signaling. On the contrary, a specific TRAF-6 knockout in myeloid cells abrogated atheroprotective IL-10 signaling and exacerbated atherosclerosis (109). These findings suggest cell-specific anti- and pro-inflammatory functions of TRAF-6. In line with this, we have recently shown that a global knockout of TRAF-6 had no effect on atherosclerosis, which could be explained by competing pro- and anti-atherosclerotic effects (108). Although inhibition of the CD40-TRAF-2/3/5 axis did not impact atherosclerosis, a knockout of TRAF-5 that was not specific for the CD40-binding site exacerbated atherosclerosis in mice by promoting leukocyte accumulation and foam cell generation in the plaque (110). Likewise, disrupting TRAF-1, a negative inhibitor of TRAF-2, protected from atherosclerosis (107), while the genetic inhibition of TRAF-2 did not change atherosclerotic lesion burden (108). The effects of CD40 signaling are challenging to resolve, because of the complex interplay of distinct upstream receptors for TRAFs and various cell-type specific effects (130). In addition, atherogenesis may also be driven by diet-induced metabolic changes, and the functional role of TRAFs could diverge in metabolism. This is best illustrated by the findings that CD40 is expressed on adipocytes and functions as pro-inflammatory stimulator (131), while T cell CD40 in the setting of dysmetabolism and adipose tissue inflammation is limiting inflammation (49), likely by a TRAF-2/3/5-dependent mechanism (132). In contrast, knocking out the CD40-TRAF-6 binding site protected from dysmetabolism and adipose tissue inflammation (132). These mechanisms may directly and indirectly impact on atherosclerotic lesion burden. A common observation, however, is the modulation of leukocyte infiltration in CD40- or TRAF-deficient animals by regulating adhesion factor expression on ECs and leukocytes (106, 107, 110, 133).

## CD40L in Thrombosis and Hemostasis

### CD40 Ligation Induces Platelet Activation and Aggregation

Beyond their classical role in hemostasis and thrombosis, platelets contribute to acute and chronic inflammatory pathologies, such as atherosclerosis (134, 135). Key mechanisms to support inflammation include adhesion of platelets to the inflamed endothelium, where they can bind leukocytes and drive their recruitment by supporting rolling and firm adhesion (135–137). Platelets can also support inflammation by releasing inflammatory cytokines and chemokines that are stored in α-granules, including CCL5, CXCL4, and IL-1β. CD40L is an immediate activation marker of platelets that is stored in α-granules and mobilized to the cell surface (138), where it can be detected by flow cytometry within seconds after stimulation *in vitro* (40, 138). Beside T cells, CD40L gene expression can be detected in megakaryocytes, the progenitors of blood platelets (Figure 2). It is unclear whether CD40L is actively transcribed in platelets. Notably, in one study *Cd40lg* transcript were detected in platelets (138). However, the more important functional regulation seems to occur by mobilization of granule stored CD40L to the cell surface. Membrane-bound CD40L is subsequently shed by MMPs, likely ADAM10, -17 (12), MMP-2 (13), and -9 (14) within minutes to hours in an integrin αIIβ3-dependent mechanism. This process is also enhanced after ligation of CD40 on platelets (40). CD40L released to the blood circulation is usually referred to as soluble CD40L (sCD40L) (139). 95% of sCD40L in the blood circulation is estimated to originate from platelets (25, 120, 140), but T cells (12) or activated ECs (26) are a possible alternative source.

(s)CD40L has been shown to be platelet agonist itself, promoting activation and aggregation of platelets, thereby driving thrombus formation, growth, and stability (54, 94, 140–142). Clinically, levels of sCD40L correlate with thrombotic events, likely reflecting enhanced platelet activation and a release of CD40L in thrombotic disease (24). Neutralizing CD40L by monoclonal antibodies in clinical trials induced an enhanced rate of thromboembolic complications in humans and primates, suggesting that CD40L is required for stabilizing thrombi (126, 127) (also see Therapeutic Inhibition of CD40L in Clinical Disease). *Cd40l^−/−^* mice showed a delayed arterial occlusion time in a thrombosis model—an effect that could be reversed by a transfusion with sCD40L (54). CD40L deficiency resulted in smaller thrombi *in vitro* (143). In addition, mice with a genetic deficiency of CD40L (144) or after treatment with a blocking anti-CD40L antibody (56, 145) show prolonged bleeding time, although it is not clear whether these effects are platelet dependent or not.

### Platelet Receptors for CD40L

The integrins αIIβ3, α5β1 (80), and its “classical” binding partner CD40 (40, 142) have been identified as platelet-expressed receptors for CD40L. It is not entirely clear whether platelet CD40L engages all three identified receptors on platelets simultaneously. Interestingly, formation and stabilization of thrombi by CD40L occurred primarily through binding of αIIβ3 and subsequent tyrosine phosphorylation of the integrin cellular domain (54, 94). These effects were absent when platelets were activated with recombinant CD40L lacking the KGD β3-recognition motif. CD40L stimulation induced αIIβ3 outside-in signaling, Akt-phosphorylation, and glycoprotein VI-induced platelet aggregation in a PI3K-dependent manner but did not require IKKα (146). Interestingly, CD40L promoted thrombus growth in the absence of CD40, suggesting that CD40L induces thrombosis and platelet aggregation *via* binding to αIIβ3 or α5β1, but not *via* CD40.

Some of CD40L’s effects may also be caused by binding to CD40, albeit experimental evidence is controversial in this regard. For instance, thrombus formation was not changed in *Cd40^−/−^* mice (54, 141) but delayed after treatment with a blocking anti-CD40L or an anti-CD40 antibody (56), as well as in *Cd40^−/−^* mice in another study (141). This discrepancy could be caused by tissue- and vessel type-specific expression of CD40 (147). Of note, CD40L induces platelet activation, the release of α- and dense granules (142), as well as aggregation of platelets (141, 148) in a CD40-dependent manner. CD40 ligation on platelets regulated expression of the platelet chemokine CXCL4 (PF4) (148). Recombinant, mutated CD40L lacking the CD40-binding site failed to induce platelet activation (141). Moreover, an inhibiting antibody to CD40 prolonged bleeding time (145). These reports, taken together, suggest that platelet CD40 is required to fully exhibit platelet activation, while αIIβ3 may be dispensable for platelet activation (142), although experimental evidence is contradicting in this regard (149). A potential explanation is that a blockade of αIIβ3 may abrogate CD40L shedding and the release of soluble CD40L (139). Subsequently, decreased levels of sCD40L would limit a possible auto-stimulation of platelets by self-expressed sCD40L (40, 142). While downstream signaling events are mediated primarily *via* TRAF-6 in atherosclerosis, Donners et al. found that mice with a genetic inhibition of CD40/TRAF-6 signaling did not alter platelet deposition and thrombus formation in an *in vitro* flow chamber assay (133), suggesting an involvement of TRAF-2/3/5 instead. Indeed, another study found CD40 ligation in platelets activates TRAF-2, the GTPase Rac1, and p38 MAPK (141).

**Figure 4:**
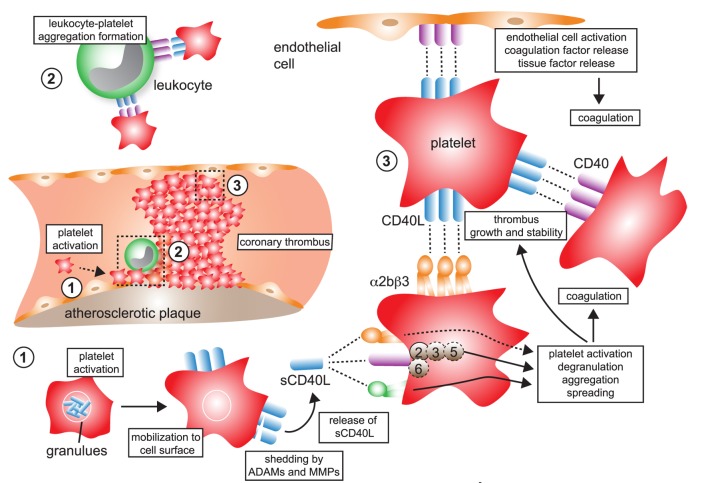
CD40L and its receptors in thrombosis and hemostasis. (1) Monomeric CD40L is stored in α- and dense granules of platelets. Upon platelet activation and degranulation, CD40L is mobilized to the cell surface and forms biologically active trimers. These are shed within minutes to hours by ADAMs and matrix metalloproteinases (MMPs) and released as monomers of soluble CD40L (sCD40L). These have been reported to form dimers and trimers in circulation that a biologically active. (2) Platelet-expressed CD40L binds to its receptors on leukocytes, α5β1 and CD40, but not αMβ2 (Mac-1), to form platelet–leukocyte aggregates. Notably, platelet-expressed CD40 can bind to CD40L-expressing leukocytes, such as activated T cells. (3) Ligation of platelet-expressed receptors, α5β1 and CD40, by CD40L initiates platelet activation cascades that eventually result in the surface expression of platelet receptors, such as P-selectin, degranulation, aggregation, and spreading. CD40 ligation on platelets activates a TRAF-2 dependent, but not TRAF-6 dependent, signaling pathway. It is a controversy whether ligation of α2bβ3 by CD40L participates in platelet activation. However, physical interactions between platelet-expressed α2bβ3 and CD40L, as well as between CD40 and CD40L, help to stabilize platelet aggregates and fuel thrombus growth. Binding of platelet CD40L and of sCD40L to its endothelial receptors CD40 and α5β1 promotes endothelial cell activation, pro-inflammatory gene expression, and secretion of coagulation factors, including tissue factor, which in turn can amplify thrombosis and coagulation.

Some newer evidence also suggests the integrin α5β1 in CD40L-mediated platelet activation: blocking antibodies against α5β1 prevented CD40L-induced platelet activation and lowered expression of P-selectin and PAC-1, the activation epitope of αIIβ3 in human platelets (149), raising the possibility that at least α5β1 and CD40 (and maybe partially αIIβ3) cooperate in CD40L-induced platelet activation. In contrast, the fourth receptor for CD40L, αMβ2 (CD11b) (56, 69), is not expressed by platelets. Some observations have raised the possibility that CD40L binding may not be exclusive to certain receptors but instead occurs in a heterotrimeric fashion, by which trimeric (surface-expressed) CD40L simultaneously binds to two or more receptors (50). The involvement of CD40L-associated pathways in thrombosis and hemostasis is summarized in Figure 4.

### Inflammatory and Immune Effects of Platelet CD40L/CD40 Signaling

It has been challenging to specifically attribute platelet-expressed CD40 and CD40L to atherosclerosis due to the limited availability of conditional knockout models in the past. However, two recent reports have addressed this question by transfusing platelets deficient for either of those molecules in competent recipients. After transferring *Cd40^−/−^Apoe^−/−^* platelets, Gerdes et al. found a twofold reduction of atherosclerosis in recipient *Apoe^−/−^* mice compared with a transfer of WT platelets. This effect was accompanied by a reduction of macrophages and neutrophils in atherosclerotic aortas in animals that received *Cd40^−/−^* platelets. The authors speculated whether this decrease of myeloid cell accumulation in the lesion was caused by an inability of CD40-deficient platelets to adhere to the endothelium and to recruit leukocytes and/or by reduced platelet–leukocyte aggregates. Indeed, it was observed in the same study that a lack of CD40 decreased platelet adhesion to the carotid endothelium and lowered formation of leukocyte–platelet aggregates (148). CD40-deficient mice also demonstrated reduced leukocyte–platelet complexes in another study (150). Formation of leukocyte–platelet aggregates—likely by a P-selectin-dependent mechanism—has previously been shown to enhance leukocyte recruitment into the atherosclerotic plaque (135–137). Also, an adoptive transfer of *Cd40l^−/−^* platelets protected from atherosclerosis when compared with WT platelets in another study (143). Taken together, both studies emphasize a role for the CD40L–CD40 interaction in the leukocytes-platelet cross talk.

In contrast, we found earlier that the CD40L/Mac-1 interaction does not mediate leukocyte–platelet aggregate formation (56), suggesting not all receptors for CD40L are fully capable to mediate firm binding between leukocytes and platelets. Beyond promoting leukocyte–platelet aggregation, Henn et al. demonstrated that platelet-expressed CD40L activates ECs and stimulated the secretion of pro-inflammatory cytokines, such as TNF-α and IL-1β, as well as to promote the expression of adhesion molecules that will ultimately support leukocyte recruitment (40, 138). Interestingly, some further studies have revealed that many of the pro-inflammatory consequences of platelet–endothelial interactions in inflammation depend on CD40 signaling (151–153).

Atherosclerosis is partially driven by adaptive immunity, which initiates and maintains a T cell response to autoantigens in the plaque and promotes the formation of autoantibodies (98). CD40L is a potent co-stimulatory molecule that is required in the T-cell-dependent activation of B cells. It is therefore fascinating to speculate whether platelet CD40L would participate in this response. Indeed, it was shown that platelet CD40L is sufficient to provide a co-stimulatory signal to drive T and B cell immunity in allograft rejection, viral, and bacterial infection (154–156). However, the specific impact of platelet CD40L in atherosclerosis-associated immunity is largely unknown.

## Mechanistic Hallmarks of CD40L

### Role of CD40L in Lymphocyte Recruitment to the Plaque

**Figure 5:**
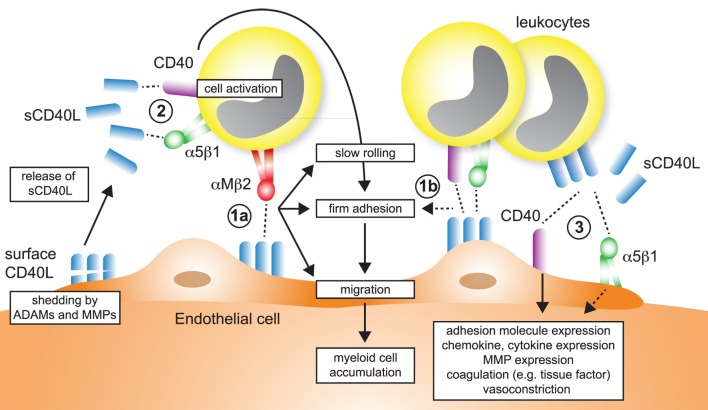
Participation of CD40L pathways in leukocyte recruitment. Several simultaneous mechanisms that contribute to the recruitment and transmigration of inflammatory leukocytes in inflamed tissue have been identified: (1a) endothelial cells (ECs) express membrane-bound CD40L in inflammation. The exact dynamics and stability of endothelial-expressed CD40L is unknown, but shedding by ADAMs and matrix metalloproteinases (MMPs) has been described. The interaction of αMβ2 (Mac-1) with endothelial CD40L mediates slow rolling and firm adhesion, which ultimately favors myeloid cell accumulation in tissues. (1b) It has been proposed that besides αMβ2, also CD40 and α5β1, expressed on leukocytes may participate in rolling and adhesion. (2) Ligation of α5β1 and CD40 on leukocytes, either by sCD40L or membrane-bound CD40L from leukocytes, platelets, or ECs activate pro-inflammatory signaling in leukocytes and increased expression of adhesion receptors that will in turn enhance cell recruitment. (3) α5β1 and CD40 expressed on ECs is ligated by sCD40L or cell-bound CD40L. As a result, ECs upregulate adhesion molecule expression and secretion of chemokines, besides other pro-inflammatory changes.

Recruitment of lymphocytes and myeloid cells initiates atherogenesis and maintains plaque inflammation (157). Several findings support that CD40L regulates leukocyte accumulation in the plaque (Figure 5), as evidenced by altered numbers of lesional macrophages, T cells, and other leukocytes after modulation of CD40L, its receptors, or signaling intermediates (Table 2). A smaller number of studies have interrogated how CD40L regulates accumulation, adhesion, and migration of leukocytes functionally. In summary, four different mechanisms can be gathered from these studies:

– CD40L is expressed on activated ECs (26, 151, 152) and functions as adhesion factor itself. We have previously shown an impairment of *Cd40l^−/−^* ECs in an *in vitro* flow chamber system independent of other adhesion factors expressed on ECs (56). Endothelial CD40L interacts with Mac-1 (56, 69) (Figure 5-1a), CD40 (158, 159), and α5β1 (86) (Figure 5-1b).
– Endothelial or circulating CD40L binds to its receptors (CD40, α5β1) on circulating, rolling, or arrested leukocytes, which activates pro-inflammatory signaling cascades in these (Figure 5-2). As a results, leukocytes upregulate adhesion molecules that are required for rolling, adhesion, or transmigration (160): ligation of monocyte CD40 by endothelial CD40L increases pro-inflammatory cytokine expression (152); circulating CD40L promotes the activation of neutrophils and integrin activation in a CD40-dependent fashion (161); and blocking CD40L abolishes leukocyte recruitment (162). CD40L furthermore activates myeloid cells by engagement of α5β1 (80). On the contrary, CD40L is a biased agonist for Mac-1 not activating outside-in signaling.
– Circulating CD40L, leukocyte-, platelet- (40, 138), or microparticle-bound CD40L (163) stimulates adhesion factor expression (44, 164), cytokine- and chemokine expression (152, 165, 166) in ECs in a CD40- (165, 167) and α5β1- (80) dependent mechanism (Figure 5-3). Activation of ECs promotes recruitment of leukocytes. Inflammatory cytokines (44) and modified lipids (168) were shown to increase expression of CD40 on ECs, explaining why CD40L-dependent activation of ECs is enhanced in the pro-inflammatory milieu of atherosclerosis.
– Circulating CD40L activates platelets or leukocytes and favors the formation of platelet–leukocyte aggregates (Figure 4-2).

### Mechanisms of CD40L-Mediated Plaque Instability

In human atherosclerosis, a stable plaque is characterized by a thick fibrous cap with intact collagen fibers and a small necrotic core, while an unstable or vulnerable plaque is characterized by thin cap, collagen breakdown, and a large necrotic core. Although mice do not develop spontaneous plaque rupture, an increased accumulation of macrophages and lipids, and lowered numbers of SMCs and collagen resemble a less stable plaque in mouse atherosclerosis (1). The complex interplay of stabilizing factors and destabilizing factors, such as expression of inflammatory cytokines and matrix-disintegrating enzymes is believed to orchestrate the composition and physical stability of the plaque (44, 169). In gene enrichment analysis, CD40-dependent signaling pathways are enriched in macrophage-rich regions of unstable human plaques (Figure 3). CD40L has been shown to modulate several facets of plaque instability: all major cell types that build the scaffold or populate the core of the atherosclerotic plaque, lymphocytes, macrophages, SMCs, and ECs, express CD40L and CD40 (26, 27, 34, 36, 159, 164, 170–172). MMPs are the main destabilizing collagenases in the plaque (169). MMPs were shown to be induced by CD40L and CD40 ligation in SMCs, ECs, and macrophages (44). CD40 in human plaques not only colocalizes with MMPs and SMCs but its ligation also upregulates the expression of MMP-1/2/3/8/9/13 and pro-inflammatory cytokines (173–176). CD40 expression in SMCs increases after stimulation with pro-inflammatory cytokines such as TNF-α, IL-1β, and INF-γ (26). CD40 ligation in SMCs enhances the expression of the adhesion molecules VCAM-1, ICAM-1, E-selectin, and the chemokine receptors CCR1, CCR5, and CXCR4, which in turn increase the recruitment of leukocytes into the plaque (44). Also, IL-1β-converting enzyme and tissue factor are secreted by SMCs in a CD40-dependent manner (177, 178). Interestingly, the interaction of CD40L to Mac-1 is not important for SMC functioning. We have recently tested a genetic knockout for CD40 in the setting of neointima formation after a surgical wire injury in the carotid artery, which induces SMC proliferation. CD40-deficient animal showed a smaller neointima, but not animals treated with the CD40L/Mac-1 blocker, M7, suggesting that CD40-dependent signaling pathways predominate in SMC activation and proliferation (150). Besides modulation of stabilizing collagen and fibrous cap formation, CD40L has also been implicated in necrotic core formation: ligation of CD40 induced proapoptotic pathways in lymphocytes and macrophages (7, 179) along with increased expressed of pro-inflammatory cytokines and collagenases (160). In addition, several studies have also suggested that the thrombotic potential of the plaque is regulated by CD40L, mainly by expression of pro-thrombotic tissue factor in tissue-resident cells, macrophages, SMCs, and ECs (160, 180–182).

### Therapeutic Inhibition of CD40L in Clinical Disease

The participation of CD40L in pro-inflammatory and autoimmune disease, such as arthritis, nephritis, organ rejection, autoimmune diabetes, inflammatory bowel disease, and SLE (28–32) has led to the clinical evaluation of antibody-mediated neutralization of CD40L in human disease. For instance, BG9588, a humanized antihuman CD40L antibody, was tested in a phase II study in 28 patients with lupus glomerulonephritis. Although treatment with BG9588 and other antihuman CD40L antibodies showed a significant reduction in disease-specific parameters (127, 183), including levels of autoantibodies and renal function, its clinical evaluation was stopped because of an increased rate of thromboembolic events in one study, where two cases of myocardial infarction were recorded (127). In addition, CD40L blockade with the anti-CD40L clone (ATTC 5C8.33) caused multiple thrombotic events in monkeys (126). On the contrary, reports on different antihuman CD40L clones and preparations (e.g., IDEC-131) have demonstrated clinical safety (184), suggesting that antibody clones and/or preparations may contribute to its side effects. Thus, the mechanism by which CD40L raised thromboembolism is controversial: many experimental studies in the mouse have supported the idea that CD40L acts as a cross-linker of platelets in spontaneous formed thrombi (54) and that its inhibition destabilizes thrombi and favors thromboembolism. However, it is unknown whether this mechanism is the cause for thromboembolic events in humans. Finally, a study employing the anti-CD40L antibody CDP7657 (a PEGylated F_ab_ fragment of the antibody) failed to induce thromboembolism in Cynomolgus monkeys, while dose-dependently inhibiting CD40L-dependent immune function (185). Likewise, a version of the antihuman CD40L antibody clone hu5c8 that does not bind to the Fc receptor was clinically effective in the same study without affecting platelet function *in vitro* (185). Both results suggest that clinical side effects of hu5c8 may be caused by binding to the Fc receptor on platelets through FcγRIIa and, therefore, in part independent of CD40L itself (186).

## Concluding Remarks

A considerable number of studies over the last two decades have demonstrated that CD40L is a powerful mediator of inflammation and thrombosis. Its functional repertoire is fine tuned by the differential expression of CD40L itself and of its receptors in various cell types. Clinically, CD40L was proposed as biomarker of atherothrombosis, while its receptor CD40 associates with cardiovascular disease in GWAS. The receptor-unspecific therapeutic inhibition of CD40L, however, is unfavorable, because CD40L is required for many physiological processes in immunity and hemostasis. Its broad functional repertoire may, however, enable to develop cell- and function specific inhibition strategies. This was best illustrated by M7, a specific inhibitor of the CD40L/Mac-1 interaction that mediates leukocyte recruitment, while not interfering with CD40 binding. Yet, little is known about the cell-type specific role of CD40L and its receptor, but the development of conditional and tissue-specific knockout models will help to clarify these in future. The emerging concept of cell- and function-specific blockade could even be expanded to specific cell signaling pathways engaged by CD40L/CD40 signaling. An interesting study by van den Berg et al. lately demonstrated the potency of a selective blocker of the CD40-TRAF-6 interaction that protected from diet-induced obesity (187). Such therapeutic strategies, along with others, have revived the search for selective tools to block CD40L in cardiovascular pathologies and may ultimately point to novel therapeutic strategies against human cardiovascular disease.

## Methods

### Differential Gene Expression

Baseline gene expression of different human immune cell types quantified by RNAseq was extracted from the Protein Expression Atlas of the European Bioinformatics Institute (EMBL-EBI) (33). Expression values were retrieved as FPKMs, underwent hierarchical clustering, and normalization as row scores by Morpheus (Broad Institute).

### Gene Set Enrichment Analysis

GenePattern 2.0 (188) was used to process RNA array data for GSEA (189), which was run with the default settings (100 iterations, weighted). Gene enrichment was tested for the core CD40-signaling gene signature (Table 3) on published transcriptomes of human stable and ruptured atherosclerotic plaques from laser microdissected macrophage-rich regions of carotid endarterectomy specimen [accession number Array Express E-GEOD-41571 (100)]. Enrichment was tested between ruptured and stable plaques. A *P*-value of 0.2 was considered significant. The expression of the enrichment-driving genes was blotted on a heatmap and colored by a row score.

**Table 3:** CD40-signaling core gene signature.

Gene symbol	Name(s)
*Akt1*	AKT serine/threonine kinase 1
*Birc2*	Baculoviral IAP repeat containing 2, CIAP1
*Birc3*	Baculoviral IAP repeat containing 3, CIAP2
*Cd40*	CD40
*Cd40lg*	CD40 ligand, CD40L, TRAP, Gp39, TNFSF5
*Chuk*	Conserved helix–loop–helix ubiquitous kinase, IκB kinase α-subunit
*Ikbkb*	Nuclear factor NF-κB inhibitor kinase β, NFKBIKB
*Ikbke*	Inhibitor of nuclear factor κB kinase subunit ε
*Ikbkg*	Inhibitor of nuclear factor κ-B kinase subunit γ, NEMO
*Jak3*	Janus kinase 3
*Jun*	Jun proto-oncogene, AP-1 transcription factor subunit, AP-1
*Map2k1*	MAPK/ERK kinase 1, MKK1, MEK1
*Map2k2*	MAPK/ERK kinase 2, MKK2, MEK2
*Map2k3*	MAPK/ERK kinase 3, MKK3, MEK3
*Map2k4*	JNK-activating kinase 1, SAPK/ERK kinase 1, MAPK/ERK kinase 4
*Map2k6*	MAPK/ERK kinase 6, SAP/ERK kinase 3
*Map2k7*	JNK-activating kinase 2, SAP/ERK kinase 4, MAPK/ERK kinase 7
*Map3k1*	MAPK/ERK kinase kinase 1, MEKK1
*Map3k14*	NF-kappa-β-inducing kinase, NIK
*Map3k7*	TGF-β activated kinase 1, TAK1
*Mapk1*	MAP kinase 1, ERK-2, p38
*Mapk11*	Mitogen-activated protein kinase P38 β, P38β, SAPK2
*Mapk12*	Mitogen-activated protein kinase P38 γ, SAPK3
*Mapk13*	Mitogen-activated protein kinase P38 δ, PRKM13
*Mapk14*	Mitogen-activated protein kinase P38 α, P38α
*Mapk3*	Mitogen-activated protein kinase 3, ERK1
*Mapk8*	C-Jun N-terminal kinase 1, JNK-1, SAPK1
*Mapk9*	C-Jun N-terminal kinase 2, JNK-2, SAPK1a
*Nfkb1*	Nuclear factor κB subunit 1, NF-κB
*Nfkbia*	NFKB inhibitor α, Iκ-Bα
*Nfkbib*	NFKB inhibitor β, Iκ-Bβ, TRIP-9
*Nfkbie*	NFKB inhibitor ε, Iκ-Bε
*Pik3ca*	Phosphatidylinositol-4,5-bisphosphate 3-kinase catalytic subunit α, PI3K
*Rela*	RELA proto-oncogene, NF-κB subunit, NFKB3, P65
*Relb*	RELB proto-oncogene, NF-κB subunit
*Stat3*	Signal transducer and activator of transcription 3, STAT3
*Tnfaip3*	TNF-α-induced protein 3, A20
*Traf1*	TNF receptor-associated factor 1, TRAF1, EBL6
*Traf2*	TNF receptor-associated factor 2, TRAF2, TRAP3
*Traf3*	TNF receptor-associated factor 3, TRAF3
*Traf3ip2*	TRAF3-interacting protein 2, nuclear factor NF-kappa-B activator 1
*Traf5*	TNF receptor-associated factor 5, RNF84
*Traf6*	TNF receptor-associated factor 6, RNF85

## Author Contributions

DW, NM, and AZ analyzed data and designed and wrote the manuscript.

## Conflict of Interest Statement

The authors declare that the research was conducted in the absence of any commercial or financial relationships that could be construed as a potential conflict of interest.
